# Pro-Inflammatory Role of AQP4 in Mice Subjected to Intrastriatal Injections of the Parkinsonogenic Toxin MPP+

**DOI:** 10.3390/cells9112418

**Published:** 2020-11-05

**Authors:** Agnete Prydz, Katja Stahl, Soulmaz Zahl, Nadia Skauli, Øivind Skare, Ole Petter Ottersen, Mahmood Amiry-Moghaddam

**Affiliations:** 1 Laboratory of Molecular Neuroscience, Division of Anatomy, Department of Molecular Medicine, Institute of Basic Medical Sciences, University of Oslo, P.O. Box 1105, Blindern, 0317 Oslo, Norway; agnpry@gmail.com (A.P.); katja.stahl@gmail.com (K.S.); soulmaz.r@gmail.com (S.Z.); nadia.skauli@medisin.uio.no (N.S.); 2 Department of Occupational Medicine and Epidemiology, National Institute of Occupational Health, Gydas vei 8, 0363 Oslo, Norway; Oivind.Skare@stami.no; 3 President’s Office, Karolinska Institutet, Nobels väg 6, 171 77 Stockholm, Sweden; ole.petter.ottersen@ki.se

**Keywords:** AQP4, Parkinson’s disease, MPP+, astrocyte, microglia, neuroinflammation

## Abstract

Aquaporin-4 (AQP4) is critically involved in brain water and volume homeostasis and has been implicated in a wide range of pathological conditions. Notably, evidence has been accrued to suggest that AQP4 plays a proinflammatory role by promoting release of astrocytic cytokines that activate microglia and other astrocytes. Neuroinflammation is a hallmark of Parkinson’s disease (PD), and we have previously shown that astrocytes in substantia nigra (SN) are enriched in AQP4 relative to cortical astrocytes, and that their complement of AQP4 is further increased following treatment with the parkinsonogenic toxin MPTP (1-methyl-4-phenyl-1,2,3,6-tetrahydropyridine). Here, we investigated the effect of *Aqp4* deletion on microglial activation in mice subjected to unilateral intrastriatal injection of 1-methyl-4-phenylpyridinium (MPP+, the toxic metabolite of MPTP). Our results show that MPP+ injections lead to a pronounced increase in the expression level of microglial activating genes in the ventral mesencephalon of wild type (WT) mice, but not *Aqp4^−/−^* mice. We also show, in WT mice, that MPP+ injections cause an upregulation of nigral AQP4 and swelling of astrocytic endfeet. These findings are consistent with the idea that AQP4 plays a pro-inflammatory role in Parkinson’s disease, secondary to the dysregulation of astrocytic volume homeostasis.

## 1. Introduction

Of the 13 mammalian aquaporins (AQP) that have been discovered to date, three are expressed and functional in brain tissue [1]. One of these (AQP1) shows a very restricted expression, limited to the choroid plexus, while the other two (AQP4 and AQP9) are found in many brain regions, including the SN [2,3]. Other AQPs, including AQP2, 3, 5, 6 and 8, have been reported to be expressed in the brain, but so far only at the mRNA level [4].

AQP4 is by far the most prevalent aquaporin in brain—being expressed across all brain regions—and is particularly abundant at the brain-blood interface as a major component of the molecular assembly in perivascular astrocytic endfeet [5,6,7]. AQP9 is more weakly expressed, but with a preponderance in the mesencephalon [3]. Thus, both AQP4 and AQP9 are relevant in the context of Parkinson’s disease.

AQP9 has a broad substrate profile and has been shown to mediate transport of parkinsonogenic compounds like arsenite and 1-methyl-4-phenylpyridinium (MPP+) [8,9]. In our previous study, we showed that the targeted deletion of AQP9 protects against MPP+ induced degeneration of nigral neurons [8]. Based on this finding, we hypothesized that AQP9 could be parkinsonogenic by acting as an influx route of compounds that are toxic to nigral dopaminergic neurons.

Unlike AQP9, AQP4 is strikingly selective for water, due to the dimensional constraint and configuration of the channel pore, and plays an important role in brain water homeostasis and dyshomeostasis, including brain edema [10,11,12,13]. However, evidence has accrued to show that AQP4 is engaged in a host of brain functions and dysfunctions that are only indirectly coupled to or even independent of water transport [7,14].

The list of neurological conditions in which AQP4 might play a pathophysiological role includes epilepsy [15,16] and neurodegenerative diseases, such as Alzheimer’s disease (AD) [17,18,19,20], amyotrophic lateral sclerosis (ALS) [21,22], Huntington’s disease (HD) [23] and spongiform encephalopathy (SE) [24]. Studies have also highlighted possible links between AQP4 and infectious and neuroinflammatory diseases, most notably meningitis [25], malaria [26] and neuromyelitis optica (NMO) [27].

Several neurological conditions—including epilepsy and a number of neurodegenerative diseases—also show hallmarks of neuroinflammation [28,29]. This is also true of Parkinson’s disease (PD) [30]. Here, we use an animal model to explore our hypothesis that AQP4 could have a proinflammatory impact in PD. If so, this would further expand the number of roles attributed to AQP4 in neurological conditions.

The present work was inspired by study of Li et al. [31], which provided evidence of a proinflammatory role of AQP4 both in a mouse model of experimental autoimmune encephalopathy (EAE) and in an in vitro model. In both experimental approaches, mice or murine cell cultures with targeted deletion of AQP4 showed lower inflammation scores than wild type (WT) mice used as controls. The in vitro model showed that lipopolysaccharide (LPS) induced release of the cytokines TNF-α and IL-6 from astrocytes in an AQP4-dependent manner, possibly as a consequence of astrocytic swelling. These cytokines are known to activate cerebral microglia during neuroinflammation [32,33].

The goal of the present study was twofold. First, we set out to investigate whether intrastriatal injections of 1-methyl-4-phenylpyridinium (MPP+)—a mouse model of PD developed in our laboratory—upregulate expression of genes involved in microglial activation. Second, if such an effect could be demonstrated, we asked whether it is amplified by targeted deletion of AQP4. An answer in the affirmative would encourage further studies to resolve whether AQP4 serves a proinflammatory role in PD.

The timelines of this study is highlighted by recent investigations on AQP4 in brain. Notably, we have shown that nigral astrocytes are enriched in AQP4 relative to cortical astrocytes, and that their complement of AQP4 is further increased following treatment with MPTP (1-methyl-4-phenyl-1,2,3,6-tetrahydropyridine) [34]. MPP+—the parkinsonogenic drug used in the present study—is the active metabolite of MPTP [35]. Further, imaging studies have revealed abnormal accumulation of water in mesencephala of patients with advanced PD [36].

Here, we demonstrate that MPP+ injections lead to an increase in microglial activation in the ventral mesencephalon, and that this increase is curbed by deletion of *Aqp4*. We also show, in WT mice, that MPP+ injections cause an upregulation of nigral AQP4 and swelling of astrocytic endfeet. These findings are consistent with the idea that upregulation of AQP4 plays a pro-inflammatory role in Parkinson’s disease—possibly secondary to dysregulation of astrocytic volume homeostasis.

## 2. Materials and Methods

### 2.1. Animals

Mixed gender and adult WT C57BL/6J mice (Jackson Laboratories, Boulder, CO, USA) bred in the local animal facility (*n* = 20) and constitutive *Aqp4*^−/−^ mice backcrossed for more than 10 generations on C57BL/6J background (*n* = 22) were used in this study. *Aqp4*^−/−^ mice, generated as described by Thrane et al. [37], carry a mutated *Aqp4* gene where exon 1−3 is replaced by a flippase recognition target (FRT)-neomycin-FRT-LoxP–validated cassette inserted downstream of exon 3, and a LoxP site inserted upstream of exon 1, thus, avoiding any expression of putative splice variants. Both experimental groups (*Aqp4*^−/−^ and WT controls) were bred and kept at the local animal facility under the same conditions. For control purposes, we also purchased WT mice with the C57BL/6J genetic background from The Jackson Laboratory (*n* = 12). These animals had to be discarded as controls, as they differed from our internal controls (WT mice bred in the local facility) in regard to cell counts and their response to MPP+ (Supplementary Figure S1). The difference between these two cohorts of presumptive genetically identical animals is potentially interesting and will be the subject of further studies. Experimental protocols were approved by The Norwegian Animal Research Authority (NARA) with license number FOTS 4012 and carried out in accordance with the European Directive 2010/63/EU.

### 2.2. Stereotaxic Surgery and Injection of MPP+

The procedure was carried out as previously described [8]. In short, the animals were anesthetized with zoletil mixture (Zoletil Forte (250 mg/mL), Rompun (20 mg/mL) and Fentanyl (50 μg/mL); 0.1 mL/10 g; intraperitoneally) prior to fixing the head to a stereotaxic frame (TSE Systems, Bad Homburg, Germany). A total of 7.5 µg MPP+ (Sigma-Aldrich, St. Louis, MO, USA) dissolved in 1 µL of 0.9% saline, or 1 µL of 0.9% saline alone, was injected into the striatum +0.6 mm anterior to bregma, +2.2 mm laterally and −3.2 mm ventrally [38] at 12 µL/hr, using a syringe pump (Kd Scientific, Holliston, MA, USA) Body temperature was maintained at 36.5 ± 0.5 °C by a heating pad (PanLab, Barcelona, Spain), and the eyes were covered with Vaseline during the procedure.

### 2.3. Post-Operative Animal Care

At 24 h post-surgery, all animals were put on a heating pad overnight and were treated with Rimadyl (0.1 mL/10 g; s.c. every 12 h) to prevent pain, and a mixture of 0.9% saline and 0.9% sucrose (s.c. every 24 h) to restore water and energy balance. The animals were fed moisture pellets to improve food and water uptake. This treatment was continued for as long as needed, until the animals were sacrificed 7 days after surgery. The animals were scored daily for their weight and clinical appearance to evaluate the post-operative condition.

### 2.4. Tissue Preparation for Light and Electron Microscopic Cytochemistry

At 7 days post-surgery, the animals (WT; *n* = 20, *Aqp4*^−/−^; *n* = 22) were deeply anesthetized with zoletil mixture (0.2 mL/10 g; i.p.), then euthanized by intra-cardial perfusion fixation with 2% ice cold dextran, followed by 15 min of 4% formaldehyde (FA), freshly depolymerized from paraformaldehyde (PFA), dissolved in 0.1 M phosphate buffer (PB) for light microscopic cytochemistry, or 4% FA and 0.1% glutaraldehyde in 0.1 M PB for electron microscopic cytochemistry (all from Sigma-Aldrich). Brains were dissected out and post-fixed for 24 h in the fixation solution.

### 2.5. Immunocytochemistry

The tissue used for light microscopic cytochemistry was cryo-protected by sequential immersions in 10%, 20% and 30% sucrose solutions. The midbrain was then isolated and sectioned into coronal sections of 40 µm on a HM 450 freeze microtome (Microm, Walldorf, Germany). From each animal, 10 sections were systematically selected for further immunohistochemical analyses (immunofluorescence; WT (*n* = 3), *Aqp4*^−/−^ (*n* = 3) and stereological analysis; WT (*n* = 7), *Aqp4*^−/−^ (*n* = 9), with a slice interval of 120 µm, covering the entire substantia nigra pars compacta (SNpc), from Bregma −2.70 to −3.80 [38]. Immunocytochemistry for stereological quantifications has been previously described [8]. In brief, free-floating sections were blocked and permeabilized before incubation in primary antibody (Table 1) overnight, followed by incubation with secondary and tertiary antibody. Finally, sections were incubated with 3,3′-diaminobenzidine (DAB; Sigma-Aldrich) dissolved in 0.1 M PBS, then treated with a DAB-solution containing 0.03% H_2_O_2_ (Applichem, Darmstadt, Germany), before washing and mounting with glycerin-gelatin.

For immunofluorescence and confocal imaging, free floating sections were blocked, permeabilized and incubated with primary antibody (Table 1), prior to incubation with species-specific secondary antibodies prior to mounting. Images were collected with a LSM 510 META Confocal Microscope (Zeiss, Jena, Germany) using a 40× oil objective. 4′,6-diamidino-2-fenylindol (DAPI) fluorescence was captured at 405 nm, Alexa 488 at 510 nm, Cy3 at 568 nm and Cy5 at 670 nm.

### 2.6. Stereological Quantifications of Dopaminergic Density in the Midbrain

Stereological quantifications of dopaminergic cells in midbrain subregions was performed bilaterally in WT and *Aqp4*^−/−^ mice injected with MPP+ or saline. The investigator was blinded to genotype and treatment. The procedure has been described in detail [8].

### 2.7. Electron Microscopy

Tissue blocks <1 mm^3^ of SNpc from both the ipsilateral and the contralateral hemisphere of WT and *Aqp4*^−/−^ mice were dissected out, cryoprotected in glycerol and quickly frozen in liquid propane (−170 °C). The tissue was then subjected to freeze substitution [39], embedded in Lowicryl HM20 Resin (Lowy, Waldkraiburg, Germany) at −30 °C and polymerized by UV light. Ultrathin sections of 90–100 nm were cut from the blocks using an ultramicrotome (Leica EM UC6, Vienna, Austria) and transferred to mesh grids [40]. Each section was contrasted with uranyl acetate and lead citrate for 1.5 min and rinsed in ddH_2_O in-between. The sections were examined using an electron microscope (Tecnai 12, FEI Company, Eindhoven, The Netherlands).

### 2.8. Electron Microscopic Analysis

For the quantification of endfoot width, as an indicator for swelling, 25 electron micrographs of randomly selected capillaries from SN of WT (*n* = 4) and *Aqp4*^−/−^ mice (*n* = 4) were obtained from each section at 26,500× magnification. Endfoot width was assessed by calculating endfoot area per unit length of adluminal membrane using the software program ImageJ (U. S. National Institutes of Health, Bethesda, MD, USA). The analyzer was blind to genotype and area.

### 2.9. RNA Isolation and Quantitative Real Time qPCR Analysis

At 7 days post-surgery, the animals injected with MPP+ (WT (*n* = 6), *Aqp4*^−/−^ (*n* = 5) or saline WT (*n* = 3), *Aqp4*^−/−^ (*n* = 3)) were decapitated under isofluorane (Baxter, Deerfield, IL, USA) anesthesia and the brains were dissected out. The brains were cut along the midsagittal line to separate the injected and control hemispheres. The midbrain and striatum were dissected out from both hemispheres, snap frozen and stored at −80 °C until analysis. The total RNA was isolated from the regional brain samples using the RNeasy Plus Mini Kit (QIAGEN, Hilden, Germany). The RNA concentration and integrity were determined using the NanoDrop 2000c spectrophotometer (Thermo Scientific, Oslo, Norway) and agarose gel electrophoresis. The cDNA was synthesized using GoScript Reverse Transcription System (Promega, Madison, WI, USA) using Oligo (dT)15. The gene expression was evaluated by using the qPCR absolute quantification. The qPCR was performed in a total volume of 20 µL, containing the Power SYBR Green PCR Master Mix (Applied Biosystems, Oslo, Norway), forward and reverse primers (10 µM), and 10 ng of total cDNA per reaction. Thermal cycling was performed on the StepOnePlus system (Applied Biosystems) with the following conditions: 95 °C for 10 min, followed by 40 cycles at 95 °C for 15 s and 60 °C for 1 min and at the end the melting curve step. No reverse transcriptase (NRT) and no template control (NTC) negative controls were included in the study. Using the NormFinder software (Aarhus, Denmark), the expression variation of *Actb*, *H2afz*, *Pgk1* and *Ubc* as endogenous reference genes were evaluated. *Ubc* was selected as the most stable endogenous control for normalization across groups and regions with a stability value of 0.184. One-way ANOVA with Bonferroni post hoc test was used for statistical analysis of the data. The data are presented as mean ± 2SE, and *p* < 0.05 was considered as significant. A list of the primer pairs used in the experiment is shown in Table 2.

## 3. Results

### 3.1. Survival Rate and Clinical Appearance Post-Surgery

All animals were scored daily for their clinical appearance, including the parameters weight loss, inactivity, appearance and reaction patterns, giving a total score ranging from 0–12. Animals reaching a score of 10 or above were considered severely affected by the toxin and were euthanized, due to ethical concerns. Among the total number of animals treated with MPP+ (*n* = 32), five animals died or were euthanized. Among those animals, three animals were WTs, while two animals were *Aqp4*^−/−^. There was no difference in behavioral assessments between WT and *Aqp4*^−/−^ mice.

### 3.2. Unilateral Intrastriatal Injections of MPP+ Lead to Astrogliosis and Microgliosis in the Ipsilateral SN

**Figure 1:**
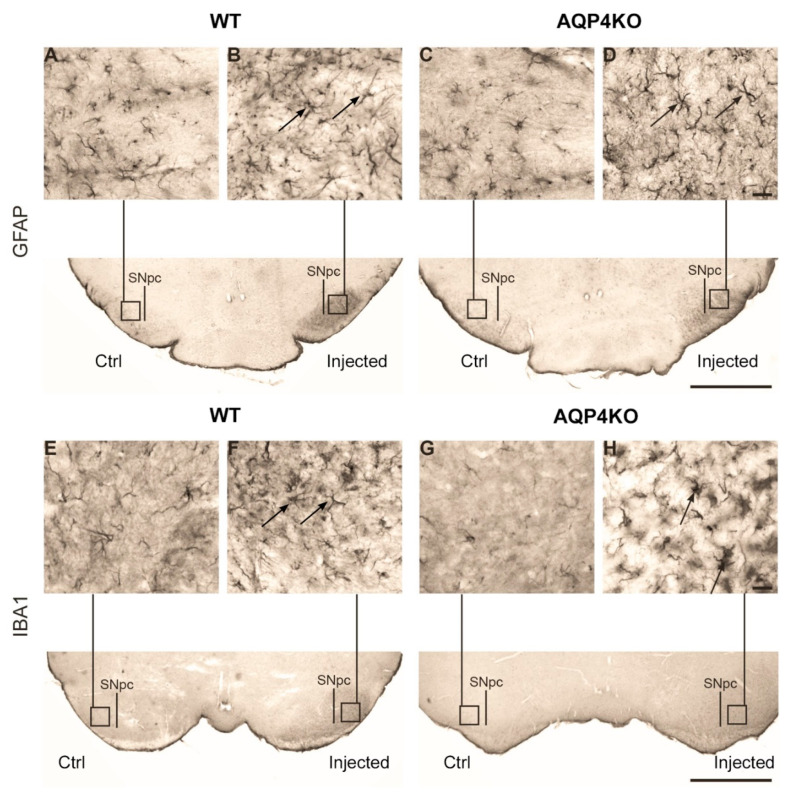
Increased expression of glial fibrillary acidic protein (GFAP) and (ionized calcium-binding adapter molecule 1) Iba1 in substantia nigra (SN) following treatment with MPP+. Representative micrographs showing midbrain sections immunostained with antibodies to the astrocyte marker GFAP upper panel (**A**–**D**) or the microglial marker Iba1 lower panel (**E**–**H**). Unilateral injection of 1-methyl-4-phenylpyridinium (MPP+) in the striatum resulted in increased expression of GFAP (arrows **B**,**D**) in the ipsilateral substantia nigra pars compacta (SNpc) (injected, **B**,**D**) compared to the contralateral hemisphere (Ctrl, **A**,**C**) in wild type (WT) (**A**,**B**) as well as in *Aqp4*^−/−^ animals (**C**,**D**). An increased expression of Iba1 (arrow **H**) is also visible in the ipsilateral SNpc (**F**), compared to the contralateral hemisphere (**E**) in WT, as well as in *Aqp4*^−/−^ animals, respectively, (**H**,**G**). Iba1 showed a slightly higher staining intensity in WT compared to *Aqp4*^−/−^ animals (lower panel, compared the area around SNpc in the injected hemispheres). Scale bar: 1000 µm; inset: 20 µm.

Astrogliosis and microgliosis have been demonstrated in the ventral midbrain of many animal models of PD, including systemic treatment with MPTP [34,41]. We investigated if unilateral intrastriatal MPP+ injections induce astrogliosis and microgliosis in the ventral midbrain. Light microscopic immunohistochemistry of midbrain sections of animals subjected to unilateral intrastriatal MPP+ injections showed stronger glial fibrillary acidic protein (GFAP) and ionized calcium-binding adapter molecule 1 (Iba1) staining in the injected hemisphere, compared to the control hemisphere (Figure 1A–H). WT and *Aqp4*^−/−^ animals did not differ in this regard. In both groups of animals, GFAP-positive astrocytes were most abundant in a distinct area corresponding to the SN of the injected hemisphere (Figure 1B,D). In both genotypes, the SN of the injected hemispheres showed strong immunostaining for Iba1, with WT mice displaying a slightly higher staining intensity than *Aqp4*^−/−^ animals (Figure 1E–H). Microglia stained for Iba1 exhibited enlarged cell bodies and chubby processes (Figure 1F,H) indicative of a microglial reactive state [42].

### 3.3. Unilateral Intrastriatal MPP+ Injections Lead to a Strong Increase in the Transcript Levels of Microglial Activating Genes in Ipsilateral Midbrain of Wild Type but Not Aqp4^−/−^ Mice

**Figure 2:**
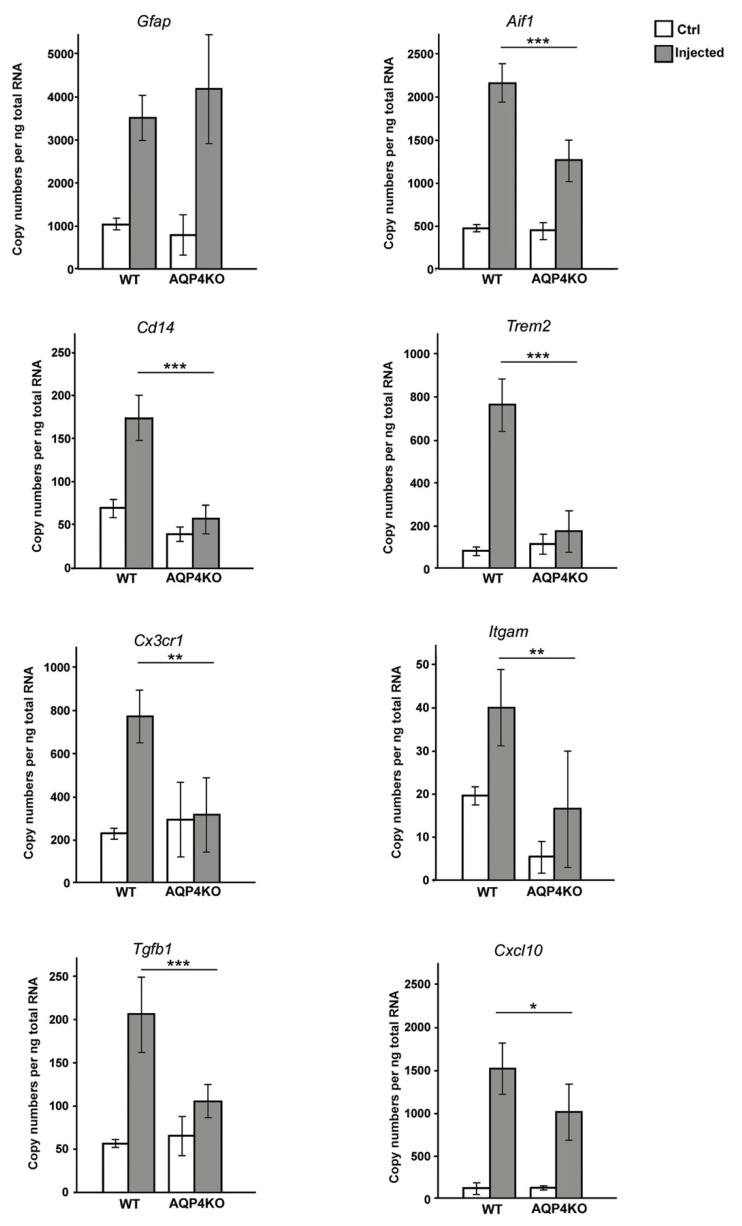
Increased mRNA levels of microglial activating genes in ipsilateral midbrain of WT but not *Aqp4*^−/−^ mice, following unilateral intrastriatal MPP+ injections. Bars are mean ± 2SEM. * *p* < 0.05, ** *p* < 0.01, *** *p* < 0.001.

We performed quantitative real time PCR analysis on samples from midbrain of injected and control hemispheres of WT and *Aqp4*^−/−^ mice (Figure 2). Confirming the immunohistochemical data, our analysis showed increased expression level for *Gfap* in the midbrain of the MPP+ injected hemisphere compared to midbrain of the control hemisphere in both WT (control: 1035 ± 134 injected: 3487 ± 542 *p* < 0.001) and *Aqp4*^−/−^ mice (control: 753 ± 448, injected: 4182 ± 1438 *p* < 0.001). There was no significant difference between the two genotypes. The expression level of the general microglial marker Iba1 (*Aif1*) was significantly increased in the injected hemisphere of both genotypes. Notably, the transcript levels of *Aif1* in the ipsilateral midbrain were significantly higher in WT than in *Aqp4*^−/−^ mice (WT: 2217.4 ± 242.2 vs *Aqp4*^−/−^: 1295.3 ± 220.8 *p* < 0.001).

To resolve whether AQP4 plays a pro-inflammatory role, we analyzed the expression levels of key genes coding for microglial membrane receptors known to be upregulated during inflammation. We specifically investigated the expression levels of *Cd14*, a key organizer of microglial inflammatory response [43]; *Trem2*, a receptor promoting microglial survival, proliferation and phagocytic activity [44]; *Cx3cr1*, a chemokine receptor involved in microglial inflammatory response [45,46]; and *Itgam*, the gene coding for the integrin receptor CD11b involved in microglial adhesion and migration [47]. In WT animals, our qPCR analysis revealed a several fold increase in the expression levels of each of these genes in midbrain samples ipsilateral to MPP+ injections. In *Aqp4*^−/−^ mice, in contrast, none of these genes differed significantly between injected and control hemispheres in regard to levels of expression (Figure 2).

We also analyzed the expression levels of the two cytokines, *Tgfb1* and *Cxcl10,* previously shown to be upregulated in MPTP models of PD [48,49]. Under normal conditions in the midbrain, TGF-β1 is mainly expressed by neurons, while under pathological conditions, it is, in addition, highly expressed in activated microglia [50], and its secretion leads to the upregulation of microglial CX3CR1 [51,52]. CXCL10 is expressed in and secreted by astrocytes and to some extent by microglia [53,54]. Earlier studies have shown that release of CXCL10 from astrocytes is involved in early microglial activation, initiating migration and inducing a pro-inflammatory phenotype [55]. Expression levels of both chemokines were strongly increased in the midbrain of the injected hemisphere of WT mice, compared to the control side (*Tgfb1*, control: 60.4 ± 6, injected: 202.9 ± 41.4, *p* < 0.001; *Cxcl10*, control: 42.8 ± 18.2, injected: 1492.7 ± 340, *p* < 0.001). However, in the *Aqp4*^−/−^ midbrain samples, only the increase in the expression level of *Cxcl10* reached significant levels (control: 83.9 ± 37.8, injected: 1128.6 ± 342, *p* < 0.001), while *Tgfb1* failed to increase significantly, following unilateral intrastriatal MPP+ injection (injected: 104.8 + 23.7, control: 66.4 + 17.6, *p* > 0.05), indicating reduced microglial activation.

Quantitative real-time PCR analysis showed a significant increase in the expression levels of *Gfap* and *Aif1* in the midbrain of MPP+ injected hemispheres in both the WT and *Aqp4*^−/−^ mice. The increase observed in the injected hemispheres of the WT mice was significantly higher for *Aif1* than the increase in the injected hemisphere of *Aqp4*^−/−^ mice (*p* < 0.01). In the case of *Gfap*, there was a significant increase in the transcript levels in the injected hemisphere compared to the control hemisphere of both genotypes *p* < 0.001, with no significant difference between the genotypes.

Expression of *Cd14* was increased from 70.2 ± 9.2 mRNA copies per ng RNA in the control midbrain to 174.5 ± 26.4 copies in the injected midbrain of the WT mice (*p* < 0.001), compared to 49.5 ± 10.4 in the control and 83.8 ± 26 in the injected hemispheres of the *Aqp4*^−/−^ mice (*p* = 1.0). The trend was similar for *Trem2* (WT control: 76.4 ± 13.6, WT injected: 568.3 ± 116, *p* < 0.001 vs *Aqp4*^−/−^ control: 111.1 ± 39.4, *Aqp4*^−/−^ injected: 222.0 ± 64, *p* = 1.0) *Cx3cr1* (WT control: 243.3 ± 40.6, WT injected: 743.9 ± 171.6, *p* < 0.001 vs *Aqp4*^−/−^ control: 296.2 ± 176.8, *Aqp4*^−/−^ injected: 315.7 ± 169.2, *p* = 1.0), *Itgam* (WT control: 20.3 ± 3, WT injected: 41.9 ± 10, *p* = 0.001 vs *Aqp4*^−/−^ control: 6.6 ± 4.6, *Aqp4*^−/−^ injected: 17.6 ± 11.2, *p* > 0.05). The mRNA expression of *Tgfb1* and *Cxcl10,* were significantly increased in the ipsilateral hemisphere of the WT animals compared to control hemisphere, and the increase was significantly higher than that observed in the *Aqp4*^−/−^ animals, at *p* < 0.05 and *p* < 0.01, respectively. In the *Aqp4*^−/−^ mice, the increase in the expression levels of *Cxcl10* and not *Tgfb1,* reached significant levels in the midbrain ipsilateral to the MPP+ injection, compared to the control midbrain.

### 3.4. Expression of AQP4 in SN after MPP+ Injections

**Figure 3:**
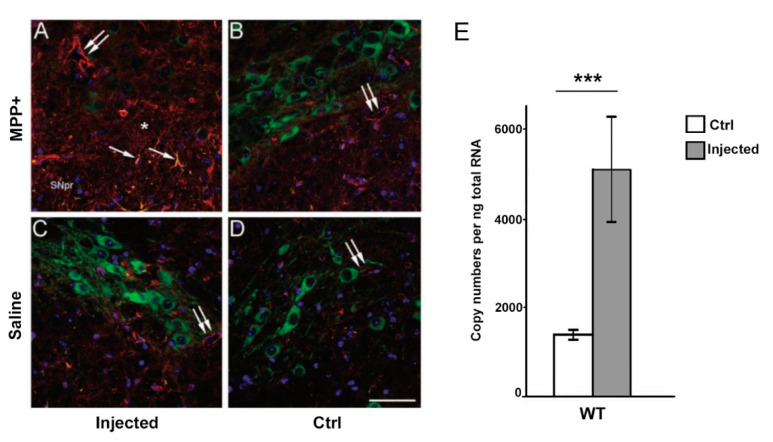
Increased expression of Aquaporin-4 (AQP4) protein and mRNA in the substantia nigra following treatment with MPP+ (**A**–**D**). Unilateral injection of MPP+ in the striatum results in loss of TH-positive cells (green) in the ipsilateral SNpc (**A**, injected) compared to the contralateral SNpc (**B**, ctrl) and animals receiving unilateral injections of saline (**C**,**D**). Strong upregulation of AQP4 (red) is further observed in the ipsilateral SN, particularly around larger vessels (double arrows) (**A**) compared to larger vessels in the control hemisphere (**B**) and saline controls (**C**,**D**). Increased AQP4 labeling is also evident in non-endfeet membranes of the neuropil (asterisk) following treatment with MPP+ (**A**). The toxin also induced upregulation of GFAP (orange) in the ipsilateral hemisphere (single arrow), (**A**). The injected hemisphere showed significantly fewer TH-positive cells (green, **A**) compared to the control hemisphere (**B**). Increased levels of *Aqp4* mRNA following treatment with MPP+ (**E**). Quantitative real-time PCR analysis showed more than two-fold higher expression of *Aqp4* mRNA in midbrain samples ipsilateral to the MPP+ injection compared to midbrain samples of the control hemisphere (ctrl). Bars are mean ± 2SEM. *** *p* < 0.001 Scale bar: 50 μm.

We have previously shown that systemic MPTP treatment causes an upregulation of AQP4 expression in SN [34]. Here, we investigated whether unilateral intrastriatal injections of MPP+ have a similar effect. Our immunofluorescence analysis revealed an increased expression of AQP4 ipsilateral to, but not contralateral to MPP+ injections, particularly around larger vessels and in the neuropil of the SN (Figure 3A,B). Ipsilateral and contralateral SN showed no difference in AQP4 immunofluorescence labeling intensity when MPP+ injections were replaced by unilateral intrastriatal injections of saline (Figure 3C,D).

Quantitative real time PCR analysis showed that *Aqp4* expression in the midbrain was significantly increased in the hemisphere treated with MPP+, as compared to the contralateral control hemisphere and saline controls (Figure 3E). The transcript levels of *Aqp4* mRNA in hemispheres treated with MPP+ was 4976 ± 1110 mRNA copies per ng RNA, compared with 1397 ± 124.4 in the control hemisphere (*p* < 0.001).

### 3.5. Electron Microscopic Analysis of Endfoot Width

**Figure 4:**
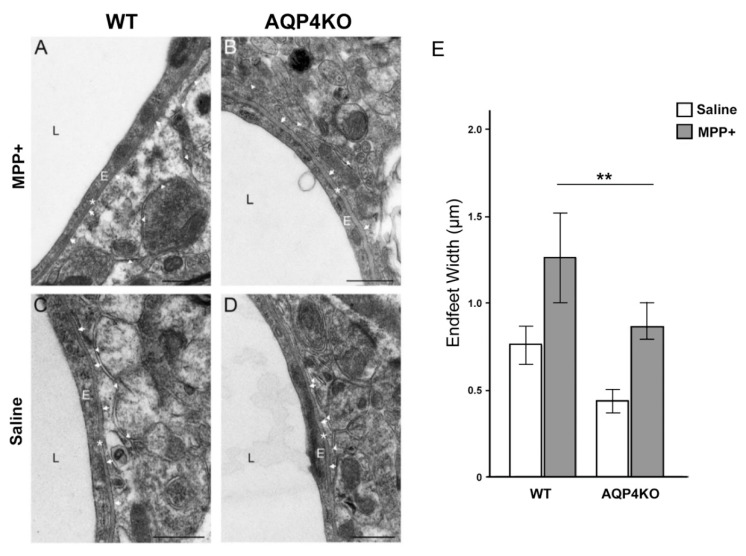
Perivascular endfoot width following treatment of MPP+ and saline. Electron micrographs of perivascular astrocytic endfeet in animals exposed to MPP+ treatment (**A**,**B**) and saline controls (**C**,**D**). Arrows mark the membrane domains of the astrocytic endfoot facing endothelial basal lamina, arrowheads mark membrane facing neuropil, thus, identifying the astrocyte endfoot. Asterisks marks basal lamina between the endothelial cell and astrocyte endfoot. The statistical analysis of the perivascular astrocytic endfoot width in SNpc of MPP+ injected hemispheres (**E**) showed a larger endfoot width in both genotypes, compared to the saline controls (*p* < 0.01). The endfoot width in the WT animals was larger compared to the *Aqp4*^−/−^ animals in both the MPP+ and saline injected hemispheres (*p* < 0.01). E, endothelial cell; L, capillary lumen. Bars are mean ± 2SEM. ** *p* < 0.01 Scale bar: 500 nm.

EM analysis showed an increased width of perivascular astrocyte endfeet in WT and *Aqp4*^−/−^ mice injected with MPP+ compared with saline controls (Figure 4). Endfoot width was larger in MPP+ injected WT mice (1.28 µm ± 0.28) than in MPP+ injected *Aqp4*^−/−^ animals (0.8 µm ± 0.2; *p* < 0.01). Additionally, after saline injections did the two genotypes differ in regard to endfoot width (Figure 4).

### 3.6. Stereological Quantification of Dopaminergic Cell Density

**Figure 5:**
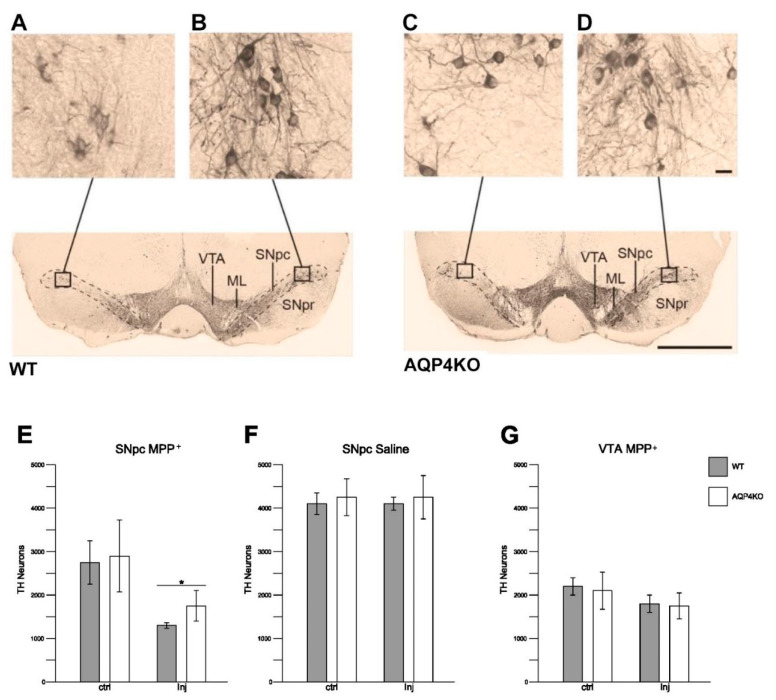
Dopaminergic neuronal cell count after unilateral treatment with MPP+ or saline in the SNpc and ventral tegmental area (VTA) of *Aqp4*^−/−^ and WT animals. Following unilateral treatment with MPP+, loss of TH-immunoreactive (TH-ir) cells is evident in the ipsilateral hemisphere (**A**,**C**) compared to the contralateral side (**B**,**D**) in both *Aqp4*^−/−^ and WT animals. In the SNpc, the average TH-ir cell count reduced from 2742 ± 490 in the control hemisphere to 1401 ± 82 in the injected hemisphere of the WT animals (*p* < 0.001) (E). In *Aqp4*^−/−^ animals, the average TH-ir cell count for the control and injected hemispheres were 2940 ± 856 and 1789 ± 262, respectively (*p* = 0.052). The number of TH-ir cells in the MPP+ injected hemisphere was significantly higher in *Aqp4*^−/−^ mice than in WT (**E**). Unilateral treatment with saline did not result in reduction in the dopaminergic cell count in the ipsilateral hemisphere of *Aqp4*^−/−^ (*p* = 0.948) or WT animals (*p* = 0.916), (**F**). There were no differences between the injected hemispheres or the control hemispheres of the two genotypes, respectively *p* = 0.732 and *p* = 0.693 (**F**). In the VTA, no significant differences were observed in the cell count of TH-positive cells in the two hemispheres of the two genotypes, (*p* = 0.607 for *Aqp4*^−/−^ and *p* = 0215 for WT, **G**). There were no differences between the genotypes in the injected side (*p* = 0.860) or the control sides (*p* = 0.864) (**G**). Bars are mean ± 2SEM. Scale bars: 1000 µm; inset: 20 µm. * *p* < 0.05.

Dopaminergic cells (TH-ir cells) in the ipsi- (Figure 5A,C) and contralateral (Figure 5B,D) hemispheres of SNpc were quantified in *Aqp4*^−/−^ and WT animals subjected to unilateral intrastriatal injections of MPP+ or saline. The number of TH-ir cells in the MPP+ injected hemisphere was significantly higher in *Aqp4*^−/−^ mice than in WT, with average cell counts of respectively 1789 ± 262 and 1401 ± 82 (*p* < 0.05, Figure 5E).

In animals treated with saline, no difference in TH-ir cell counts was observed between genotypes or between the ipsi- and contralateral side within genotypes (Figure 5F).

TH-ir cell counts in ventral tegmental area (VTA) revealed no significant differences between ipsi- and contralateral midbrain following unilateral MPP+ injections. Further, cell counts in VTA did not differ between WT and *Aqp4*^−/−^ mice after MPP+ injections, nor after injections of saline (Figure 5G).

## 4. Discussion

The question explored in the present study is whether AQP4—the most prevalent aquaporin in brain—is involved in the pathophysiology of PD, and if so—is it protective or does it exacerbate disease progression?

Recent studies in our laboratory and others have shown that AQP4 is more strongly expressed in nigral astrocytes than in astrocytes in cortex or hippocampus, and that its expression is further increased after MPTP injections in mice [34], and after 6-hydroxydopamin (6-OHDA) injections in rats [56]. It is also interesting to note that imaging studies have revealed abnormal accumulation of water in mesencephala of patients with advanced Parkinson’s disease [36]. Further, several studies have pointed to a putative coupling between AQP4 and inflammation [31,57]. These findings prompted us to investigate whether AQP4 could be involved in neuroinflammatory processes relevant for the development of PD. We chose a model that was successfully used to identify a putative link between AQP9 and loss of dopaminergic neurons. Our results show that AQP4 deletion restrains MPP+ induced upregulation of genes involved in microglia activation. Specifically, compared with WT mice, mice with targeted deletion of *Aqp4* revealed significantly lower expression of microglial genes known to be involved in microglial migration and phagocytosis.

Under pathological conditions, microglial activation is triggered by signal substances and inflammatory mediators released by peripheral immune cells, damaged neurons and astrocytes [32,33,58]. Of these cell types, only astrocytes are known to express AQP4. Thus, it is likely that the effects of *Aqp4* deletion presently reported are mediated by astrocytes.

Neuroinflammation is a hallmark of PD and of animal models of PD [59]. Microglia have been shown to be the main actors in the neuroinflammatory process contributing to dopaminergic cell loss, and several lines of evidence indicate that reactive astrocytes play an essential role in microglia activation. Upon exposure to neuronal α-synuclein, astrocytes express high levels of proinflammatory cytokines involved in migration and activation of microglia [53,60]. Moreover, the key role of astrocytes in MPTP-induced neuroinflammation and microglial activation was demonstrated in a recent study where astrocyte-specific deletion of inhibitor of nuclear factor kappa-B kinase subunit β (IKK2), a central upstream activator of NF-kB, led to reduced neuroinflammation and microglial activation following MPTP treatment [61]. These findings are in line with the idea that astrocytes may sustain neuroinflammation in PD models.

While a proinflammatory role of AQP4 is consistent with the early study of Li et al. [23], the present findings are not easily reconciled with recent studies suggesting that AQP4 may play an immunosuppressive role in models of MPTP and LPS induced neuroinflammation [62,63]. The latter studies reported that *Aqp4*^−/−^ mice treated with MPTP or LPS revealed an increased microglial inflammatory response and a more pronounced loss of TH-neurons in the SN, compared with WT mice. The *Aqp4*^−/−^ mouse line used in these studies was found to have an impaired blood brain barrier, even in the absence of any experimental intervention [64]. A leaky blood brain barrier implies that the microenvironment of neurons and glia is perturbed and that neural cells are exposed to immune cells and molecules that are normally retained in blood. This makes results difficult to interpret. The *Aqp4*^−/−^ mouse line used in the present study has an intact blood brain barrier [65]. It is worth noting that MPTP alone does not lead to a disruption of blood brain barrier function [66].

As to the mechanisms underlying the present findings, Li et al. found that release of inflammatory mediators such as interferon gamma (IFNγ) and IL-1β by astrocytes was coupled to AQP4-dependent astrocyte swelling [31]. We have previously shown that astrocytes release ATP, another activator of microglia, in an AQP4-dependent manner [37,67]. The exact mechanism of this AQP4-dependent ATP release is not known, but it could occur as part of the regulatory volume decrease where AQP4 and other AQPs have been shown to be involved [68,69].

In the present study, we demonstrate that *Aqp4*^−/−^ mice subjected to MPP+ injections show less swelling of astrocytic endfeet than MPP+ injected WT mice. We conclude that the most salient explanation of our findings is that *Aqp4* deletion curbs swelling induced release of compounds that normally cause microglial activation and inflammation.

Importantly, we show, in the current study, that astrocytic endfeet in SN are more swollen than endfeet in *Aqp4*^−/−^ mice both under basic conditions and after MPP+ exposure. The enrichment of AQP4 in nigral astrocytes and its further upregulation after toxin exposure may contribute to stronger inflammatory response and represent an additional factor contributing to the selective vulnerability of SN to toxins. Upregulation of AQP4 similar to that found here has been reported in several studies of chronic neurodegenerative diseases, including PD [34], AD [17,18,20,70], ALS [21,22] and SE [24], indicating that increased expression of AQP4 is a common feature of neurodegeneration. Interestingly the sensitivity to MPTP (and inflammatory response) is increased in aged mice [71], which express higher levels of AQP4 than younger mice [72].

The present study was designed to assess the effect of *Aqp4* deletion on microglial activation in ventral mesencephalon, a process thought to occur upstream of cell degeneration. However, even at this early time point, the loss of dopaminergic neurons after MPP+ injections was less pronounced in *Aqp4*^−/−^ mice, compared with WT controls (38% vs 45%). Our data are consistent with the findings by other groups showing that dopaminergic cell loss is lower in TLR4-deficient mice vs WT one week after treatment with MPTP (34% vs 45%) [73]. TLR4 is member of Toll-like receptor family that mediates microglial activation in neurodegenerative diseases, including PD [74].

## 5. Conclusions

It is well established that PD is associated with neuroinflammation through elevation of cytokines [75], some of which are released by reactive astrocytes [53,60] and lead to activation of microglia [76].

This is the first study to show that *Aqp4* deletion reduces microglial activation indicating that AQP4 plays a proinflammatory role in a model of PD. The mechanisms underlying this effect remain to be identified, but the most likely explanation is that AQP4 promotes swelling of astrocytes and hence swelling-induced release of ATP and other compounds that, in turn, activate nigral microglia. Taken together with previous studies, our data suggest that two of the three aquaporins that are functional in brain—i.e., AQP4 and AQP9—are implicated in PD, albeit in fundamentally different ways. While AQP9 may serve to facilitate toxin access [8], AQP4 is likely to exacerbate the effect of toxin exposure. We hypothesize that differences in AQP4 or AQP9 expression—related to age, environmental exposure or genetic factors—may affect vulnerability to PD. This hypothesis will be explored in future studies.

## Figures and Tables

**Table 1:** Primary secondary and tertiary antibodies for immunocytochemistry and -fluorescence.

Methods	Primary Antibody	Secondary Antibody	Tertiary Antibody
Immunocytochemistry	Rabbit anti-onized calcium-binding adapter molecule 1, Iba1, 1:200, Wako	Biotinylated anti-rabbit IgG (H + L) produced in goat, 1:100, Vector, Burlingame	Streptavidin-Biotinylated horse radish peroxidase complex, 1:100, GE Healthcare
	Mouse anti-glial fibrillary acidic protein, GFAP, 1:1000, Nordic BioSite AB	Biotinylated anti-mouse IgG (H + L) produced in goat, 1:100, Vector, Burlingame	Streptavidin-Biotinylated horse radish peroxidase complex, 1:100, GE Healthcare
	Mouse anti-tyrosine hydroxylase, 1:1000, Chemicon	Biotinylated anti-mouse IgG (H + L) produced in goat, 1:100, Vector, Burlingame	Streptavidin-Biotinylated horse radish peroxidase complex, 1:100, GE Healthcare
Immunofluorescence	Mouse anti-tyrosine hydroxylase, 1:1000, Chemicon	Alexa 488, 1:500, Thermo Fisher Scientific	
	Rabbit anti-AQP4, 1:400, Sigma-Aldrich	Cy3 anti-rabbit, 1:500, Jackson ImmunoResearch Laboratories	
	Chicken anti-glial fibrillary acidic protein, GFAP, 1:1000, Nordic BioSite AB	Cy5 anti-chicken, 1:500, Jackson ImmunoResearch Laboratories	

**Table 2:** Primer pairs.

Gene Name	Forward Primer	Reverse Primer	Product Size	PCR Efficiency
*Aqp4*	AGCAATTGGATTTTCCGTTG	TGAGCTCCACATCAGGACAG	203 bp	96%
*Aif1*	CTGCCAGCCTAAGACAACCA	GGAATTGCTTGTTGATCCCCT	128 bp	99%
*Cx3cr1*	TGCTCAGGACCTCACCATGTC	CTCAAGGCCAGGTTCAGGAG	246 bp	98%
*Itgam*	TGCGCGAAGGAGATATCCAG	GCCTGCGTGTGTTGTTCTTT	108 bp	94%
*Cd14*	CAGAGAACACCACCGCTGTA	CACGCTCCATGGTCGGTAGA	97 bp	96%
*Cxcl10*	ATGACGGGCCAGTGAGAATG	TCGTGGCAATGATCTCAACAC	80 bp	97%
*Tgfb1*	AATTCCTGGCGTTACCTTGG	AGTGAGCGCTGAATCGAAAG	139 bp	100%
*Trem2*	CTGGAGGACCCTCTAGATGAC	CCACAGGATGAAACCTGCCT	116 bp	98%
*Gfap*	GCACTCAATACGAGGCAGTG	GCTCTAGGGACTCGTTCGTG	207 bp	97%
*Ubc*	CGTCGAGCCCAGTGTTACCACCAAGAAGG	CCCCCATCACACCCAAGAACAAGCACAAG	112 bp	92%

## Supplementary Materials

The following are available online at https://www.mdpi.com/2073-4409/9/11/2418/s1, Figure S1: Stereological Quantification of Dopaminergic Cell Density comparing the wild type (WT) mice bred in the local facility (WT-INT) and the WT mice purchased from Jacksons (WT-EXT).

## References

[1] Nagelhus E.A., Ottersen O.P. (2013). Physiological roles of Aquaporin-4 in brain. Physiol. Rev..

[2] Mylonakou M.N., Petersen P.H., Rinvik E., Rojek A., Valdimarsdottir E., Zelenin S., Zeuthen T., Nielsen S., Ottersen O.P., Amiry-Moghaddam M. (2009). Analysis of mice with targeted deletion of AQP9 gene provides conclusive evidence for expression of AQP9 in neurons. J. Neurosci. Res..

[3] Badaut J., Petit J.-M., Brunet J.-F., Magistretti P.J., Charriaut-Marlangue C., Regli L. (2004). Distribution of Aquaporin 9 in the adult rat brain: Preferential expression in catecholaminergic neurons and in glial cells. Neuroscience.

[4] Badaut J., Lasbennes F., Magistretti P.J., Regli L. (2002). Aquaporins in brain: Distribution, physiology, and pathophysiology. Br. J. Pharmacol..

[5] Nielsen S., Nagelhus E.A., Amiry-Moghaddam M., Bourque C., Agre P., Ottersen O.P. (1997). Specialized membrane domains for water transport in glial cells: High-resolution immunogold cytochemistry of aquaporin-4 in rat brain. J. Neurosci..

[6] Amiry-Moghaddam M., Frydenlund D., Ottersen O. (2004). Anchoring of aquaporin-4 in brain: Molecular mechanisms and implications for the physiology and pathophysiology of water transport. Neuroscience.

[7] Amiry-Moghaddam M., Ottersen O.P. (2003). The molecular basis of water transport in the brain. Nat. Rev. Neurosci..

[8] Stahl K., Rahmani S., Prydz A., Skauli N., Macaulay N., Mylonakou M.N., Torp R., Skare O., Berg T., Leergaard T.B. (2018). Targeted deletion of the aquaglyceroporin AQP9 is protective in a mouse model of Parkinson’s disease. PLoS ONE.

[9] Carbrey J.M., Gorelick-Feldman D.A., Kozono D., Praetorius J., Nielsen S., Agre P. (2003). Aquaglyceroporin AQP9: Solute permeation and metabolic control of expression in liver. Proc. Natl. Acad. Sci. USA.

[10] Manley G.T., Fujimura M., Ma T., Noshita N., Filiz F., Bollen A.W., Chan P., Verkman A. (2000). Aquaporin-4 deletion in mice reduces brain edema after acute water intoxication and ischemic stroke. Nat. Med..

[11] Vajda Z., Pedersen M., Füchtbauer E.-M., Wertz K., Stødkilde-Jørgensen H., Sulyok E., Dóczi T., Neely J.D., Agre P., Frokiaer J. (2002). Delayed onset of brain edema and mislocalization of aquaporin-4 in dystrophin-null transgenic mice. Proc. Natl. Acad. Sci. USA.

[12] Frydenlund D.S., Bhardwaj A., Otsuka T., Mylonakou M.N., Yasumura T., Davidson K.G.V., Zeynalov E., Skare O., Laake P., Haug F.-M. (2006). Temporary loss of perivascular aquaporin-4 in neocortex after transient middle cerebral artery occlusion in mice. Proc. Natl. Acad. Sci. USA.

[13] Hirt L., Ternon A., Price M., Mastour N. (2008). Protective role of early aquaporin 4 induction against postischemic edema formation. Br. J. Pharmacol..

[14] Papadopoulos M.C., Verkman A.S. (2013). Aquaporin water channels in the nervous system. Nat. Rev. Neurosci..

[15] Alvestad S., Hammer J., Hoddevik E.H., Skare O., Sonnewald U., Amiry-Moghaddam M., Ottersen O.P. (2013). Mislocalization of AQP4 precedes chronic seizures in the kainate model of temporal lobe epilepsy. Epilepsy Res..

[16] Eid T., Lee T.-S.W., Thomas M.J., Amiry-Moghaddam M., Bjørnsen L.P., Spencer D.D., Agre P., Ottersen O.P., De Lanerolle N.C. (2005). Loss of perivascular aquaporin 4 may underlie deficient water and K + homeostasis in the human epileptogenic hippocampus. Proc. Natl. Acad. Sci. USA.

[17] Yang J., Lunde L.L., Paworn N., Tomohiro O., Camassa L.M.A., Nilsson N.G., Lannfelt L., Xu Y., Amiry-Moghaddam M., Ottersen O.P. (2011). Loss of astrocyte polarization in the tg-ArcSwe mouse model of Alzheimer’s disease. J. Alzheimers Dis..

[18] Pérez E., Barrachina M., Rodríguez A., Torrejón-Escribano B., Boada M., Hernández I., Sánchez M., Ferrer I. (2007). Aquaporin expression in the cerebral cortex is increased at early stages of Alzheimer disease. Brain Res..

[19] Moftakhar P., Lynch M.D., Pomakian J.L., Vinters H.V. (2010). Aquaporin expression in the brains of patients with or without cerebral amyloid angiopathy. J. Neuropathol. Exp. Neurol..

[20] Hoshi A., Yamamoto T., Shimizu K., Ugawa Y., Nishizawa M., Takahashi H., Kakita A. (2012). Characteristics of aquaporin expression surrounding senile plaques and cerebral amyloid angiopathy in Alzheimer disease. J. Neuropathol. Exp. Neurol..

[21] Kaiser M., Maletzki I., Hülsmann S., Holtmann B., Schulz-Schaeffer W., Kirchhoff F., Bähr M., Neusch C. (2006). Progressive loss of a glial potassium channel (KCNJ10) in the spinal cord of the SOD1 (G93A) transgenic mouse model of amyotrophic lateral sclerosis. J. Neurochem..

[22] Nicaise C., Soyfoo M.S., Authelet M., De Decker R., Bataveljić D., Delporte C., Roland P. (2009). Aquaporin-4 overexpression in rat ALS model. Anat. Rec. Adv. Integr. Anat. Evol. Biol..

[23] Wu T.-T., Su F.-J., Feng Y.-Q., Liu B., Li M.-Y., Liang F.-Y., Li G., Li X.-J., Zhang Y., Cai Z.-Q. (2019). Mesenchymal stem cells alleviate AQP-4-dependent glymphatic dysfunction and improve brain distribution of antisense oligonucleotides in BACHD mice. STEM CELLS.

[24] Costa C., Tortosa R., Rodriguez A., Ferrer I., Torres J.M., Bassols A., Pumarola M.B. (2007). Aquaporin 1 and aquaporin 4 overexpression in bovine spongiform encephalopathy in a transgenic murine model and in cattle field cases. Brain Res..

[25] Papadopoulos M.C., Verkman A.S. (2005). Aquaporin-4 gene disruption in mice reduces brain swelling and mortality in pneumococcal meningitis. J. Biol. Chem..

[26] Promeneur D., Lunde L.K., Amiry-Moghaddam M., Agre P. (2012). Protective role of brain water channel AQP4 in murine cerebral malaria. Proc. Natl. Acad. Sci. USA.

[27] Lennon V.A., Kryzer T.J., Pittock S.J., Verkman A.S., Hinson S.R. (2005). IgG marker of optic-spinal multiple sclerosis binds to the aquaporin-4 water channel. J. Exp. Med..

[28] Gershen L.D., Zanotti-Fregonara P., Dustin I.H., Liow J.-S., Hirvonen J., Kreisl W.C., Jenko K.J., Inati S.K., Fujita M., Morse C.L. (2015). Neuroinflammation in temporal lobe epilepsy measured using positron emission tomographic imaging of translocator protein. JAMA Neurol..

[29] Amor S., Puentes F., Baker D., Van Der Valk P. (2010). Inflammation in neurodegenerative diseases. Immunology.

[30] Troncoso-Escudero P., Parra A., Nassif M., Vidal R.L. (2018). Outside in: Unraveling the role of neuroinflammation in the progression of Parkinson’s disease. Front. Neurol..

[31] Li L., Zhang H., Varrin-Doyer M., Zamvil S.S., Verkman A.S. (2011). Proinflammatory role of aquaporin-4 in autoimmune neuroinflammation. FASEB J..

[32] Martinez F.O., Gordon S. (2014). The M1 and M2 paradigm of macrophage activation: Time for reassessment. F1000Prime Rep..

[33] Nau G.J., Richmond J.F.L., Schlesinger A., Jennings E.G., Lander E.S., Young R.A. (2002). Human macrophage activation programs induced by bacterial pathogens. Proc. Natl. Acad. Sci. USA.

[34] Prydz A., Stahl K., Puchades M., Davarpaneh N., Nadeem M., Ottersen O.P., Gundersen V., Amiry-Moghaddam M. (2017). Subcellular expression of aquaporin-4 in substantia nigra of normal and MPTP-treated mice. Neuroscience.

[35] Dauer W., Przedborski S. (2003). Parkinson’s disease: Mechanisms and models. Neuron.

[36] Ofori E., Pasternak O., Planetta P.J., Burciu R., Snyder A., Febo M., Golde T.E., Okun M.S., Vaillancourt D.E. (2015). Increased free water in the substantia nigra of Parkinson’s disease: A single-site and multi-site study. Neurobiol. Aging.

[37] Thrane A.S., Rappold P.M., Fujita T., Torres A., Bekar L.K., Takano T., Peng W., Wang F., Thrane V.R., Enger R. (2011). Critical role of aquaporin-4 (AQP4) in astrocytic Ca_2_+ signaling events elicited by cerebral edema. Proc. Natl. Acad. Sci. USA.

[38] Paxinos G.A.F.K. (2007). The Mouse Brain in Sterotaxic Coordinates.

[39] Van Lookeren Campagne M., Oestreicher A.B., Buma P., Verkleij A.J., Gispen W.H. (1991). Ultrastructural localization of adrenocorticotrophic hormone and the phosphoprotein B-50/growth-associated protein 43 in freeze-substituted, Lowicryl HM20-embedded mesencephalic central gray substance of the rat. Neuroscience.

[40] Amiry-Moghaddam M., Lindland H., Zelenin S., Roberg B.Å., Gundersen B.B., Petersen P., Rinvik E., Torgner I.A., Ottersen O.P. (2005). Brain mitochondria contain aquaporin water channels: Evidence for the expression of a short AQP9 isoform in the inner mitochondrial membrane. FASEB J..

[41] Wu D.C., Jackson-Lewis V., Vila M., Tieu K., Teismann P., Vadseth C., Choi D.-K., Ischiropoulos H., Przedborski S. (2002). Blockade of microglial activation is neuroprotective in the 1-Methyl-4-Phenyl-1,2,3,6-tetrahydropyridine mouse model of Parkinson disease. J. Neurosci..

[42] Davis E., Foster T., Thomas W. (1994). Cellular forms and functions of brain microglia. Brain Res. Bull..

[43] Janova H., Böttcher C., Holtman I.R., Regen T., Van Rossum D., Götz A., Ernst A.-S., Fritsche C., Gertig U., Saiepour N. (2015). CD14 is a key organizer of microglial responses to CNS infection and injury. Glia.

[44] Cignarella F., Filipello F., Bollman B., Cantoni C., Locca A., Mikesell R., Manis M., Ibrahim A., Deng L., Benitez B.A. (2020). TREM2 activation on microglia promotes myelin debris clearance and remyelination in a model of multiple sclerosis. Acta Neuropathol..

[45] Chen G., Zhou Z., Sha W., Wang L., Yan F., Yang X., Qin X., Wu M., Li D., Tian S. (2020). A novel CX3CR1 inhibitor AZD8797 facilitates early recovery of rat acute spinal cord injury by inhibiting inflammation and apoptosis. Int. J. Mol. Med..

[46] Ewolf Y., Eyona S., Ekim K.-W., Jung S. (2013). Microglia, seen from the CX3CR1 angle. Front. Cell. Neurosci..

[47] Jeetle J.K., Hagger G.N., Topps S.S., Male D., Rezaie P. (2002). Microglial colonization of the developing mouse brain: The effect of CD11b deletion. Neuropathol. Appl. Neurobiol..

[48] Kalkonde Y.V., Morgan W.W., Sigala J., Maffi S.K., Condello C., Kuziel W., Ahuja S.S., Ahuja S.K. (2007). Chemokines in the MPTP model of Parkinson’s disease: Absence of CCL2 and its receptor CCR2 does not protect against striatal neurodegeneration. Brain Res..

[49] Xue X., Zhang W., Zhu J., Chen X., Zhou S., Xu Z., Hu G., Su C. (2019). Aquaporin-4 deficiency reduces TGF-β1 in mouse midbrains and exacerbates pathology in experimental Parkinson’s disease. J. Cell. Mol. Med..

[50] Lehrmann E., Kiefer R., Finsen B., Diemer N.H., Zimmer J., Hartung H.P. (1995). Cytokines in cerebral ischemia: Expression of transforming growth factor beta-1 (TGF-β1) mRNA in the postischemic adult rat hippocampus. Exp. Neurol..

[51] Spittau B. (2015). Transforming growth factor β1-mediated anti-inflammation slows progression of midbrain dopaminergic neurodegeneration in Parkinson’s disease?. Neural Regen. Res..

[52] Chen S., Luo D., Streit W.J., Harrison J.K. (2002). TGF-beta1 upregulates CX3CR1 expression and inhibits fractalkine-stimulated signaling in rat microglia. J. Neuroimmunol..

[53] Farina C., Aloisi F., Meinl E. (2007). Astrocytes are active players in cerebral innate immunity. Trends Immunol..

[54] Guedes J.R., Lao T., Cardoso A.L., El Khoury J. (2018). Roles of microglial and monocyte chemokines and their receptors in regulating Alzheimer’s disease-associated amyloid-β and Tau pathologies. Front. Neurol..

[55] Clarner T.G., Janssen K., Nellessen L., Stangel M., Skripuletz T., Krauspe B., Hess F.-M., Denecke B., Beutner C., Linnartz-Gerlach B. (2015). CXCL10 triggers early microglial activation in the cuprizone model. J. Immunol..

[56] Dong Y., Yuan Y., Fang Y., Zheng T., Du D., Gao D., Du J., Liu L., He Q. (2020). Effect of aquaporin 4 protein overexpression in nigrostriatal system on development of Parkinson’s disease. Int. J. Neurosci..

[57] Tourdias T., Mori N., Dragonu I., Cassagno N., Boiziau C., Aussudre J., Brochet B., Moonen C.T., Klaus P., Dousset V. (2011). Differential aquaporin 4 expression during edema build-up and resolution phases of brain inflammation. J. Neuroinflamm..

[58] Edwards J.P., Zhang X., Frauwirth K.A., Mosser D.M. (2006). Biochemical and functional characterization of three activated macrophage populations. J. Leukoc. Biol..

[59] Vivekanantham S., Shah S., Dewji R., Dewji A., Khatri C., Ologunde R. (2015). Neuroinflammation in Parkinson’s disease: Role in neurodegeneration and tissue repair. Int. J. Neurosci..

[60] Lee H.-J., Suk J.-E., Patrick C., Bae E.-J., Cho J.-H., Rho S., Hwang D., Masliah E., Lee S.-J. (2010). Direct transfer of α-synuclein from neuron to astroglia causes inflammatory responses in synucleinopathies. J. Biol. Chem..

[61] Hammond S.L., Bantle C.M., Popichak K.A., Wright K.A., Thompson D., Forero C., Kirkley K.S., Damale P.U., Chong E.K.P., Tjalkens R.B. (2020). NF-κB signaling in astrocytes modulates brain inflammation and neuronal injury following sequential exposure to manganese and MPTP during development and Aging. Toxicol. Sci..

[62] Zhang J., Yang B., Sun H., Zhou Y., Liu M., Ding J., Fang F., Fan Y., Hu G. (2016). Aquaporin-4 deficiency diminishes the differential degeneration of midbrain dopaminergic neurons in experimental Parkinson’s disease. Neurosci. Lett..

[63] Liang R., Yong S., Huang X., Kong H., Hu G., Fan Y. (2016). Aquaporin-4 mediates the suppressive effect of Lipopolysaccharide on hippocampal neurogenesis. Neuroimmunomodulation.

[64] Zhou J., Kong H., Hua X., Xiao M., Ding J., Hu G. (2008). Altered blood–brain barrier integrity in adult aquaporin-4 knockout mice. NeuroReport.

[65] Eilert-Olsen M., Haj-Yasein N.N., Vindedal G.F., Enger R., Gundersen G.A., Hoddevik E.H., Petersen P.H., Haug F.-M.S., Skare O., Adams M.E. (2011). Deletion of aquaporin-4 changes the perivascular glial protein scaffold without disrupting the brain endothelial barrier. Glia.

[66] García-Domínguez I., Veselá K., García-Revilla J., Carrillo-Jiménez A., Roca-Ceballos M.A., Santiago M., De Pablos R.M., Venero J.L. (2018). Peripheral inflammation enhances microglia response and nigral dopaminergic cell death in an in vivo MPTP model of Parkinson’s disease. Front. Cell. Neurosci..

[67] Koizumi S., Shigemoto-Mogami Y., Nasu-Tada K., Shinozaki Y., Ohsawa K., Tsuda M., Joshi B.V., Jacobson K.A., Kohsaka S., Inoue K. (2007). UDP acting at P2Y6 receptors is a mediator of microglial phagocytosis. Nat. Cell Biol..

[68] Pizzoni A., Bazzi Z., Di Giusto G., Alvarez C.L., Rivarola V., Capurro C., Schwarzbaum P.J., Ford P. (2020). Release of ATP by TRPV4 activation is dependent upon the expression of AQP2 in renal cells. J. Cell. Physiol..

[69] Benfenati V., Caprini M., Dovizio M., Mylonakou M.N., Ferroni S., Ottersen O.P., Amiry-Moghaddam M. (2011). An aquaporin-4/transient receptor potential vanilloid 4 (AQP4/TRPV4) complex is essential for cell-volume control in astrocytes. Proc. Natl. Acad. Sci. USA.

[70] Meshorer E., Biton I.E., Ben-Shaul Y., Ben-Ari S., Assaf Y., Soreq H., Cohen Y. (2005). Chronic cholinergic imbalances promote brain diffusion and transport abnormalities. FASEB J..

[71] Gupta M., Gupta B., Thomas R., Bruemmer V., Sladek J., Felten D. (1986). Aged mice are more sensitive to 1-methyl-4-phenyl-1,2,3,6-tetrahydropyridine treatment than young adults. Neurosci. Lett..

[72] Gupta R., Kanungo M. (2011). Glial molecular alterations with mouse brain development and aging: Up-regulation of the Kir4.1 and aquaporin-4. AGE.

[73] Noelker C., Morel L., Lescot T., Osterloh A., Alvarez-Fischer D., Breloer M., Henze C., Depboylu C., Skrzydelski D., Michel P.P. (2013). Toll like receptor 4 mediates cell death in a mouse MPTP model of Parkinson disease. Sci. Rep..

[74] Fiebich B.L., Batista C.R.A., Saliba S.W., Yousif N.M., De Oliveira A.C.P. (2018). Role of microglia TLRs in neurodegeneration. Front. Cell. Neurosci..

[75] Mogi M., Harada M., Kondo T., Riederer P., Inagaki H., Minami M., Nagatsu T. (1994). Interleukin-1 beta, interleukin-6, epidermal growth factor and transforming growth factor-alpha are elevated in the brain from parkinsonian patients. Neurosci. Lett..

[76] McGeer P.L., Kawamata T., Walker D.G., Akiyama H., Tooyama I., McGeer E.G. (1993). Microglia in degenerative neurological disease. Glia.

