# Supplementary material for: Pro-Inflammatory Role of AQP4 in Mice Subjected to Intrastriatal Injections of the Parkinsonogenic Toxin MPP+
Source: Cells. 2020 Nov 5;9(11):2418. doi: 10.3390/cells9112418 (PMC7694382; doi:10.3390/cells9112418)
Supplement: Supplementary file 1 [file cells-09-02418-s001.pdf]

Supplementary data.

Figure S1.

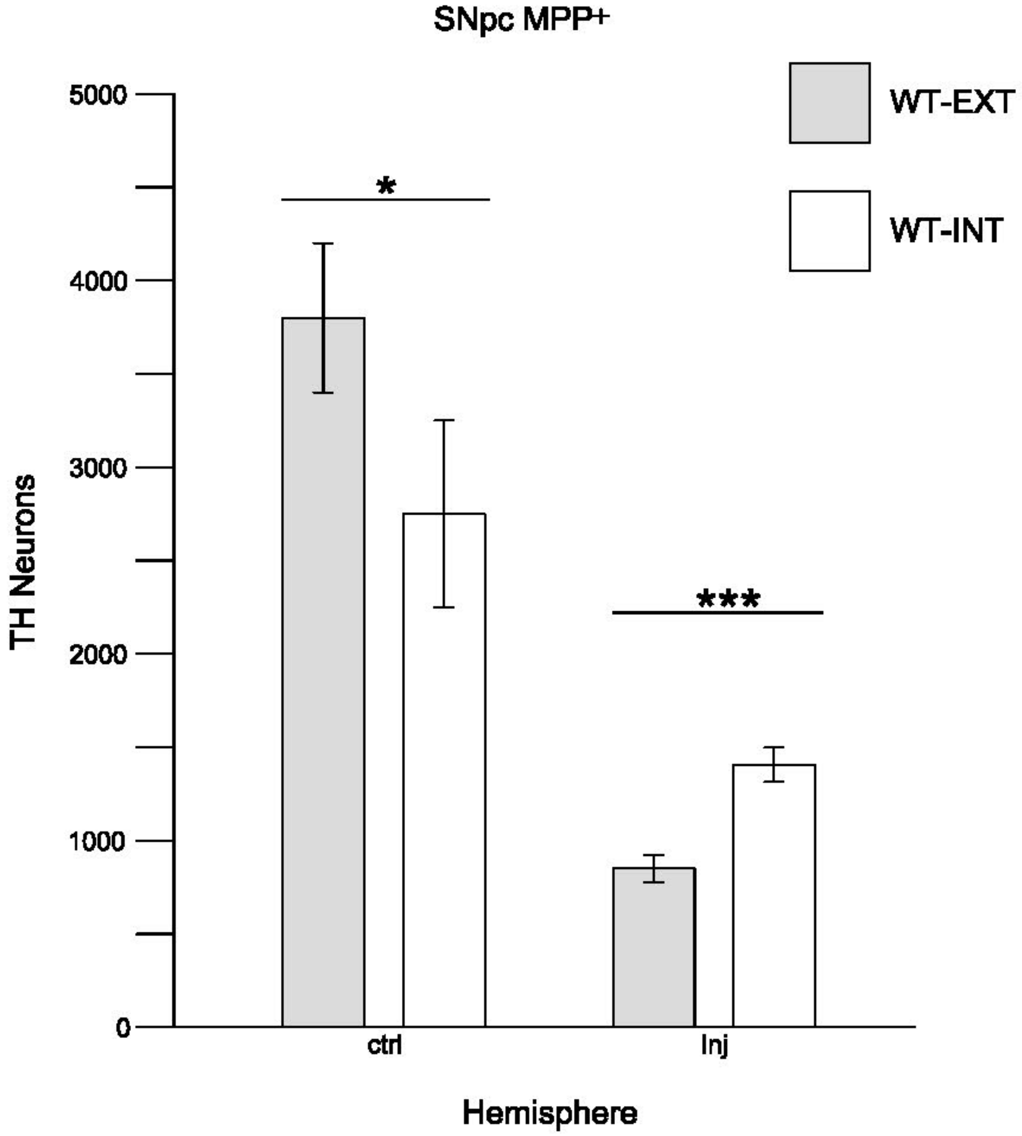

1.1. *Figure S1: Stereological Quantification of Dopaminergic Cell Density comparing WT-INT and WT-EXT animals*

The number of dopaminergic cells (TH-ir cells) in the ipsi-and contralateral hemispheres of SNpc in WT animals (WT-INT ($n = 7$); WT-EXT ($n = 5$)) were quantified by means of stereology for animals treated with a unilateral intrastriatal injection of MPP. When comparing the number of TH-ir cells in the injected hemisphere, WT-EXT showed significantly higher loss of TH-ir cells compared to WT-INT mice, with an average total cell count of respectively 863 ± 78 and 1401 ± 82 in the injected hemisphere, with $p < 0.001$. In the control hemisphere, WT-EXT showed a higher number of TH-ir cells compared to WT-INT, with an average total cell count of respectively 3822 ± 600 and 2742 ± 490, with $p < 0.05$. Numbers are Mean ± 2SEM.

2.1. Behavioral Assessments

Baseline motoric function was quantified pre-surgery using a cylinder test for paw preference. None of the animals showed differences in paw preference when tested for motoric function one day prior to surgery, regardless of genotype. The cylinder test quantifies paw preference after unilateral lesions in the nigrostriatal system, where lesions induce a preference for the ipsilateral paw [54]. No difference was observed between the genotypes post-surgery. Animals were tested for ipsilateral rotation behavior after systemic treatment with the dopamine agonist apomorphine at day 6. Significant differences were observed between animals treated with MPP+ compared to saline controls for both genotypes, but not between the genotypes after toxin-treatment. Animals were further tested for spontaneous rotational behavior in the absence of apomorphin, as the unilateral intrastriatal MPP+ injection induced ipsiversive deviated posture with spontaneous circling. No significant difference was found in net rotation between *Aqp4*$^{-/-}$ mice and WTs. Data not shown.
